# Systematic Analysis of MYB Family Genes in Potato and Their Multiple Roles in Development and Stress Responses

**DOI:** 10.3390/biom9080317

**Published:** 2019-07-30

**Authors:** Xiaoxu Li, Cun Guo, Salman Ahmad, Qi Wang, Jing Yu, Cheng Liu, Yongfeng Guo

**Affiliations:** 1 Key Laboratory for Tobacco Gene Resources, Tobacco Research Institute, Chinese Academy of Agricultural Sciences, Qingdao 266101, China; 2 Technology Center, China Tobacco Hunan Industrial Co., Ltd., Changsha 410007, China; 3 Graduate School of Chinese Academy of Agricultural Sciences, Beijing 100081, China

**Keywords:** potato, MYB, biotic stress, abiotic stress, leaf senescence

## Abstract

The MYB proteins represent a large family of transcription factors and play important roles in development, senescence, and stress responses in plants. In the current study, 233 MYB transcription factor-encoding genes were identified and analyzed in the potato genome, including 119 R1-MYB, 112 R2R3-MYB, and two R1R2R3-MYB members. R2R3-MYB is the most abundant MYB subclass and potato R2R3-MYB members together with their *Arabidopsis* homologs were divided into 35 well-supported subgroups as the result of phylogenetic analyses. Analyses on gene structure and protein motif revealed that members from the same subgroup shared similar exon/intron and motif organization, further supporting the results of phylogenetic analyses. Evolution of the potato MYB family was studied via syntenic analysis. Forty-one pairs of *StMYB* genes were predicted to have arisen from tandem or segmental duplication events, which played important roles in the expansion of the *StMYB* family. Expression profiling revealed that the *StMYB* genes were expressed in various tissues and several *StMYB* genes were identified to be induced by different stress conditions. Notably, *StMYB030* was found to act as the homolog of *AtMYB44* and was significantly up-regulated by salt and drought stress treatments. Furthermore, overexpression of *StMYB030* in *Arabidopsis* enhanced salt stress tolerance of transgenic plants. The results from this study provided information for further functional analysis and for crop improvements through genetic manipulation of these *StMYB* genes.

## 1. Introduction

Transcription factors act as key regulatory components by switching the expression of genes involved in various biological processes [1]. The MYB family has been reported to be one of the largest transcription factor families in plants, regulating various developmental processes and stress responses [2,3]. MYB transcription factors harbor a conserved MYB domain at the N terminus, generally composed of one to four imperfect repeats, each possessing approximately 52 amino acids and forming three α-helices structures. The second and third helices are often organized into a helix-turn-helix structure with three regularly spaced tryptophan residues, which play an important role in DNA binding [3,4]. Based on the number of adjacent repeats within the MYB domain, MYB proteins are classified into four groups, namely 1R-MYB, R2R3-MYB, R1R2R3-MYB, and 4R-MYB members containing one, two, three, and four MYB repeats, respectively. R2R3-MYB members are the most common subgroup of MYB members found in plants while in animals R1R2R3-MYB proteins are the predominant group [2,3]. It has been suggested that R2R3-MYB proteins have probably evolved from R1R2R3-MYB progenitors due to the loss of the R1 repeat [5].

In plants, the R2R3-MYB subfamily consists of majority of the MYB proteins and members in this subfamily play central roles in controlling plant-specific processes including plant development, secondary metabolism, and biotic/abiotic stress responses [3,6,7]. In *Arabidopsis*, the R2R3-MYB proteins have been classified into 25 subgroups and a number of *R2R3-MYB* genes have been characterized to be involved in various developmental processes [3]. In subgroup 19, AtMYB21 and AtMYB24 control anther development, while AtMYB33 and AtMYB65 in subgroup18 facilitate both anther and pollen development [8,9,10,11]. AtMYB37/RAX1, AtMYB38/RAX2, and AtMYB84/RAX3 in subgroup 14 function in axillary meristem development as partially redundant regulators whereas AtMYB68 from the same subgroup specifically regulates root growth, influencing the whole plant development under harsh conditions [12]. In subgroup 15, AtMYB0 and AtMYB23 contribute to the origination of trichome in shoots while AtMYB23 acts in root hair patterning [13,14]. Being the last developmental process, leaf senescence could be triggered by age, phytohormones, and biotic/abiotic stresses while a large number of transcription factors, including members from the NAC, WRKY, and MYB family members, were reported to participate in leaf senescence [15]. In a previous study, *AtMYB2* from subgroup 20 was found to be up-regulated during late stages of plant development and contributed to the regulation of whole plant senescence [16].

In addition, several R2R3-MYB members have been reported to be involved in regulation of metabolism in *Arabidopsis* [7]. In subgroup 7, AtMYB11/PFG2, AtMYB12/PFG1, and AtMYB111/PFG3 participate in biosynthesis of flavonols in all tissues [17]. In subgroup 6, AtMYB75/PAP1, AtMYB90/PAP2, and AtMYB113 regulate the biosynthesis of anthocyanin in vegetative tissues [18,19]. In subgroup 21, AtMYB52, AtMYB54, and AtMYB69 were suggested to be involved in biosynthesis of lignin, xylan, and cellulose [20,21]. Furthermore, in subgroup 12, AtMYB34/ATR1, AtMYB51/HIG1, and AtMYB122 control camalexin and indolic glucosinolates production in roots and late phase rosette leaves [22,23].

Furthermore, a large number of R2R3-MYB proteins were found to confer abiotic and/or biotic stress responses in *Arabidopsis* [6]. In subgroup 1, AtMYB60 and AtMYB96 control stomatal movement, drought stress, and disease resistance via the ABA signaling cascade while AtMYB30 confers a hypersensitive cell death response to disease attacks [24,25,26,27]. Similarly, AtMYB15 in subgroup 2, AtMYB33 in subgroup18, and AtMYB44 in subgroup 22 function in ABA-mediated responses to abiotic stresses and environmental signals [28,29,30,31]. In subgroup 20, AtMYB2 regulates plant response to dehydration and salinity stress after treating with exogenous ABA, AtMYB62 acts in phosphate starvation, while AtMYB108 was reported to be involved in responses to various biotic and abiotic stresses [32,33,34,35]. In defense response, AtMYB102 from subgroup 11 regulates resistance against herbivory and takes part in the response of *Arabidopsis* to aphid infestation, while AtMYB72 is a key regulator in activating induced systematic resistance upon colonization of roots by beneficial microorganisms [36,37,38].

Potato is an important food commodity worldwide, and the yield of potato is under constant threats induced by various stresses. The roles of MYB members in development and responses to biotic and abiotic stresses signify them to be potentially exploited in crop improvement. In addition to *Arabidopsis*, *MYB* family genes have been identified in a number of plant species, including tomato, grapes, rice, maize, chinese pear, sesame, and *Tamarix hispida* [39,40,41,42,43,44,45]. However, limited information is available about *MYB* family genes in potato. In the current study, a comprehensive analysis was carried out and the results revealed that the potato MYB family members may play multiple roles in potato development and in responses to various stresses.

## 2. Materials and Methods 

### 2.1. Identification and Phylogenetic Analysis of Potato MYB Proteins

The potato genome data (Release 3.4) and the *Arabidopsis* genome data were retrieved from the Sol Genomics Network (SGN, http://solgenomics.net/, Boyce Thompson Institute for Plant Research, NY, USA) and TAIR (Phoenix Bioinformatics Corporation, Fremont, CA, USA), respectively. The previously reported *Arabidopsis* MYB full-length protein sequences were used as queries to carry out BLASTP searches against the potato annotation database under the E-value cutoff of 0.1. The resulted sequences were then subjected to Pfam (https://pfam.xfam.org/, European Molecular Biology Laboratory, Cambridgeshire, UK) and SMART (http://smart.embl.de/, biobyte solutions GmbH, Heidelberg, Germany) analyses to detect the presence and number of the MYB domain. The resulted genes were named based on their physical locations across the 12 potato chromosomes. The ProtParam online toolkits (http://au.expasy.org/tools/protparam.html, Bioinformatics Resource Portal, Lausanne, Switzerland) were used to predict molecular weight and isoelectric point of candidate proteins.

The full-length protein sequences and MYB domain sequences of newly identified potato R2R3-MYB members together with the previously reported *Arabidopsis* R2R3-MYB members were subjected to perform multiple sequence alignments using MAFFT with the default settings. The alignment result of the R2R3-MYB domain was visualized by Texshade. A neighbor-joining (NJ) tree was constructed based on the prior full-length sequences alignment result using the MEGA package 6.06 with the following parameters: Poisson correction, 1000 replicate bootstrap values, and pairwise deletion. The tree file was visualized with FigTree 1.4.0 (University of Maryland, College Park, MD, USA).

### 2.2. Motif and Gene Structure Analysis

MEME (multiple expectation maximization for motif elicitation, http://meme-suite.org/, National Institutes of Health, Bethesda, MD, USA) was adopted for the identification of conserved motifs with the following parameters: Distribution of motif occurrences, zero or one per sequence; maximum number of motifs, 20; and the optimum width of each motif, between 6 and 100 residues [46].

The gene structure of *Arabidopsis* and potato *R2R3-MYB* genes were plotted by the gene structure display server (GSDS: http://gsds.cbi.pku.edu.cn, Beijing, China) by comparing the coding sequence (CDS) and genomic sequence obtained from the TAIR and SGN database, respectively [47].

### 2.3. Chromosomal Localization and Duplication Event Analysis

The chromosomal locations of the potato *MYB* genes were obtained from the SGN database and illustrated by Perl. The tandem gene duplications were identified as previously described [48]. The MCScanX program was used to identify segmental duplications based on the previous reports and the results were visualized by Circos [43]. To explore the synteny relationship of the orthologous genes obtained from potato and other tested plant species, the syntenic analysis maps were generated by the Systeny Plotter of Tbtools (South China Agricultural University, Guangzhou, China), as reported previously [49]. The non-synonymous (ka) and synonymous (ks) substitutions of each duplicated gene pair were calculated by DnaSP v5 (Universitat de Barcelona, Barcelona, Spain) [50].

### 2.4. Expression Profiling of Potato MYB Genes

The RNA-seq data of *StMYB* genes were retrieved from the International Potato Genome Sequencing Consortium (PGSC, http://solanaceae.plantbiology.msu.edu/, Michigan State University, MI, USA) that were generated in a previous study [51]. The expression data of root, stem, shoot apex, leaf, stolon, flower, and tuber were selected. The relative expression ratios of biotic and abiotic stress treatments were calculated relative to their controls, respectively. The *StMYB* gene expression data were normalized and visualized by R.

### 2.5. Growth and Stress Treatments of Potato Plants

Shoot cultures of potato Cultivar GN2 were used in this study. Shoots were inoculated and cultured on MS solid-media via nodal cutting. Potato seedlings were maintained in a growth chamber at 22 °C under 16 h light with 8 h dark for two weeks. For salt stress treatments, the seedlings were treated with 150 mM NaCl, as previously reported [48]. Further, these cultures were transferred into the soil in the same growth chamber for two months to obtain tuber. Different tissues, including shoot, shoot tip, root, young leaves, senescent leaves, and tuber were used in exploring the tissue-specific expression patterns. All of these tested samples were frozen by the liquid nitrogen and transferred to −80 °C immediately.

### 2.6. RNA Extraction and qRT-PCR Analysis

Total RNA of tested sample was extracted by Trizol, in combination with RNase-free DNase treatment. Total RNA was used for the synthesis of first strand of cDNA by using the PrimeScript™ RT reagent Kit (TaKaRa, Shiga, Japan) according to the manufacturer’s instructions. The RT-qPCR was performed on an ABI 7500 real-time PCR instrument (Applied Biosystems, Waltham, MA, USA). The potato *Elongation Factor 1-α* (*EF1α*) gene was adopted as the internal control [52]. RT-qPCR of each gene was performed with three biological replications and the resulting data were collected and analyzed by the 2^−ΔΔCT^ method. The primer sequences used in the current study were listed in Supplementary Table S1.

### 2.7. Subcellular Localization

The CDS of *StMYB030* without stop codon and the GFP fragment were amplified and inserted into the pCHF3 vector by Infusion (Invitrogen) separately, generating the *StMYB030-GFP* fusion fragment driven by the CaMV-35S promoter. The recombinant vector was introduced into *Agrobacterium* strain GV3101 for transient expression in the leaf of *Nicotiana benthamiana*. After incubation of 2–4 days, these leaves were subjected to 4,6-diamidino-2-phenylindole (DAPI) staining to confirm the position of the nucleus. As previous studies, the confocal microscope (TCS-SP8 Leica, Wetzlar, Germany) was used to capture the fluorescence signals [53,54].

### 2.8. Overexpression Analysis

The coding sequence of *StMYB030* gene was amplified and ligated into the pCHF3 by Infusion (Invitrogen, Carlsbad, CA, USA), which was under the control of the CaMV-35S promoter. This recombinant construct was introduced into *Agrobacterium* strain GV3101 and transformed into *Arabidopsis* Col-0 plants by the floral dip method [55]. The positive transgenic lines were screened out on half MS solid plates with 50 mg/L kanamycin. The T3 generation lines were generated and selected for further analysis. The *Arabidopsis* wildtype Col-0 and two *StMYB030*-overexpression transgenic lines were sown on half MS solid plates and grown vertically for seven days. Then, these seedlings were transferred to new half MS solid plates supplemented with 0 or 100 mM NaCl. All plants were grown under continuous light at 22 °C. The primary root length was measured at 14 days after treatment. Three independent biological replicates each containing five plants were conducted for each treatment. The *t*-tests were performed with GraphPad Prism 5 (GraphPad Software Inc., San Diego, CA, USA).

## 3. Results

### 3.1. Identification of MYB Genes in Potato

To identify *MYB* genes in potato, the BLASTP search was performed using the full-length sequences of the reported *Arabidopsis* MYB members as queries. Then, the resulted hits were subjected to Pfam and SMART analyses, surveying the presence and the repeat number of complete MYB motif in each candidate. As a result, a total of 233 *MYB* genes were identified, including 119 R1-MYB, 112 R2R3-MYB, and 2 R1R2R3 MYB members. No 4R-MYB member was detected in potato. We were able to map 230 of the newly identified *MYB* genes on the 12 chromosomes of potato and named them with the prefix ‘St’ implying *Solanum tuberosum*, followed by numbers in the order of physical position from top to bottom of each chromosome, starting from chromosome 1. Three of the *MYB* genes were not mapped to any chromosome and were named *StMYB231*, *StMYB232*, and *StMYB233*, respectively.

The detailed information, including length of CDS, protein molecular weight (MW), and isoelectric point (pI) were analyzed. Among the 233 identified StMYB members, StMYB009 was found to be the smallest MYB protein, whereas the largest MYB protein was StMYB209 with more than 1000 aa. The molecular weight of the identified members ranged from 7.4 to 113.4 kDa, whereas the pI ranged from 4.23 to 10.27. The detailed information could be available in Supplementary Table S2.

### 3.2. Multiple Sequence Alignment and Phylogenetic Analysis

**Figure 1:**
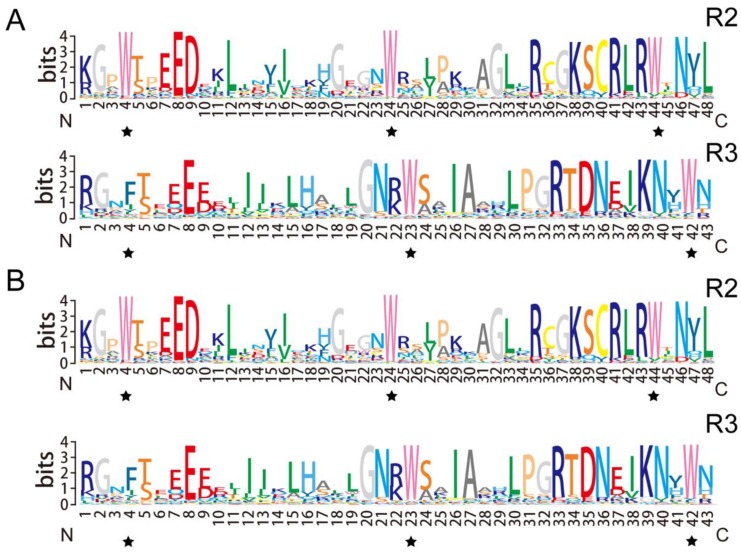
Sequence logos of the R2 and R3 MYB repeats of MYB members from potato (**A**) and *Arabidopsis* (**B**). The results were generated by multiple alignment analysis of *Arabidopsis* and potato R2R3-MYB members and visualized by Texshade. The asterisks indicate the typical conserved Trp residues in the MYB domain.

In the plant kingdom, the R2R3-MYB is the largest subgroup of MYB proteins that harbors a highly conserved DNA-binding domain composed of two adjacent MYB repeats [5]. To explore the characteristics of the potato R2R3-MYB members, the MYB domain of the potato R2R3-MYB members were extracted and subjected to multiple sequence alignment. These results revealed that the potato R2 and R3 MYB domains harbor the conserved amino acid residues, particularly the characteristic Trp. Three highly conserved Trp residues were observed in the R2 MYB domain. In the potato R3 MYB domain, only the second and third Trp were found to be conserved, whereas the first Trp was substituted by Phe or Ile (Figure 1A). In addition, the sequence features of the R2R3-MYB domain were highly conserved between the potato and *Arabidopsis* members (Figure 1B). 

**Figure 2:**
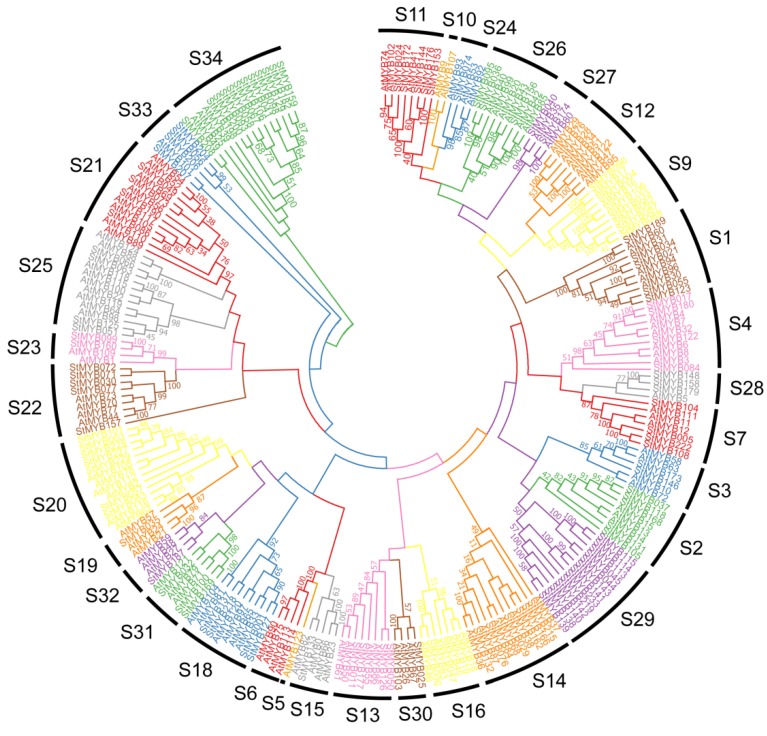
Phylogenetic analysis of potato R2R3-MYB family members. The phylogenetic tree was generated from the alignment of potato and *Arabidopsis* R2R3-MYB proteins with 1000 bootstrap replicates using the neighbor-joining (NJ) method. The potato R2R3-MYB members together with their *Arabidopsis* homologs were classified into 34 subgroups.

R2R3-MYB members are the most common subgroup of MYB members found in plants [2,3]. The multiple sequence alignment of 112 potato R2R3-MYB members and their *Arabidopsis* homologs was performed and generated a neighbor-joining tree based on the alignment (Figure 2). The results showed that all the R2R3-MYB members from potato and *Arabidopsis* fell into 34 subgroups, of which S1 to S25 subgroups were highly consistent with the previous reports and were named based on the earlier systems [3]. The rest of the subgroups were named from S26 to S34, which contained some of the Arabidopsis R2R3-MYB members that were originally not included in the 25 subgroups. It is worth mentioning that most of the subgroups possessed members from both potato and *Arabidopsis*, indicating that the expansion of R2R3-MYB members may appear before the divergence of potato and *Arabidopsis*. Intriguingly, it was found that Subgroup 29 only harbored R2R3-MYB members from potato and Subgroup 12 only contained members from *Arabidopsis*.

### 3.3. Gene Structure and Motif Composition

The gene structure could provide evolutional information. To gain insights into the evolution of the R2R3-MYB family in potato, the number and arrangement of intron-exon were identified in 112 potato *R2R3-MYB* genes and their *Arabidopsis* homologs. The results showed that genes within the same group usually have the similar intro–exon organization, and that the *StR2R3-MYB* genes shared the similar gene structures with *Arabidopsis* members in the same group (Supplementary Figure S1). Among *StR2R3-MYB* genes, the number of introns varied from 0 to 10, with the majority genes having two introns (66%) or one intron (21%). Three introns were found in four *StMYB* genes whereas 6, 9, and 10 introns were found in *StMYB208*, *StMYB063*, and *StMYB066*, respectively. Furthermore, six *StMYB* genes were found to be intron-less.

The conserved motifs of StMYB proteins were predicted by the MEME program and a total of 20 distinct motifs were identified. The motifs were named motif 1–20, among which motif 3, 4, 1, and 2 together formed the R2R3-MYB domain (Supplementary Figures S1 and S2). The potato R2R3-MYB members were featured with two conserved MYB domain repeats, motifs 3/4 and motif 1/2 were identified as the R2 MYB repeat and the R3 MYB repeat respectively, while motif 1 may be substituted by motif 8 or 17. Inconsistent with the results of phylogenetic analysis, StMYB members within the same group were found to share the similar motif organizations. Notably, motif 14 was only found in subgroup 20, motif 15 was conserved in subgroup 1, and motif 20 was unique in subgroup 29. These unique motifs may contribute to functional divergence.

### 3.4. Syntenic Analysis 

**Figure 3:**
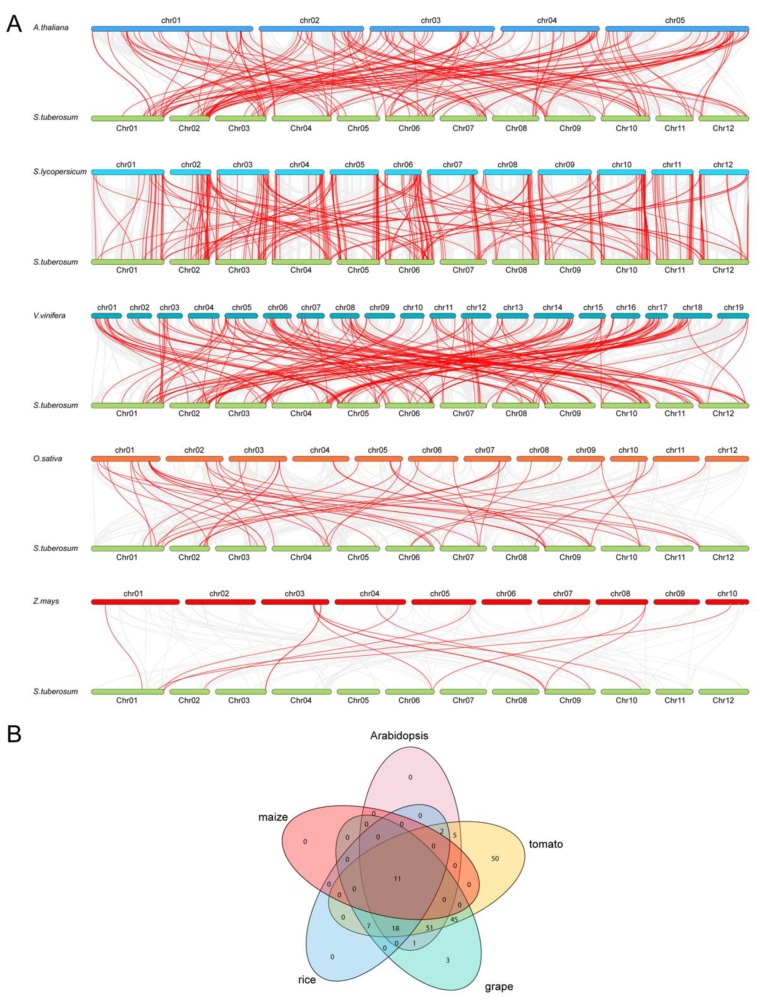
Synteny analysis of *MYB* genes between potato and five other representative species. (**A**) The gray line in the background represented the collinear blocks between potato and five other representative species, while the red line exhibited the syntenic *MYB* gene pairs. (**B**) The *MYB* genes formed the syntenic pairs between potato and all the other five selected species, which was visualized by the Venn plot.

To further explore evolution of the potato MYB family members, syntenic analysis was performed between potato and five other plant species, including tomato, grape, and *Arabidopsis* representing dicot species, while rice and maize represented monocot species (Figure 3A). The results revealed syntenic relationship between 189 of the *StMYB* genes with *MYB* genes in tomato, followed by 136 *StMYB* genes with grape, 88 *StMYB* genes with *Arabidopsis*, 29 and 14 *StMYB* genes with *MYB* genes from maize and rice, respectively. Notably, more than half of the predicted collinear pairs between potato and tomato, both belonging to the Solanaceae family, are localized at highly conserved syntenic regions comprising more than 100 syntenic genes, whereas all of the predicted collinear pairs between potato and maize were found in syntenic blocks harboring less than 10 pairs. A total of 11 *StMYB* genes were predicted to form collinear pairs with genes from all of the other five species, suggesting that these genes may have existed before the divergence of these plant species (Figure 3B). Interestingly, 69 collinear pairs identified between potato and tomato/grape/*Arabidopsis* were not found with rice/maize, implying those pairs may have appeared after the divergence of dicot and monocot species. Notably, 25 *AtMYB* genes were predicted to be paired with at least two potato genes, suggesting that these additional potato genes may have arisen from duplication events and may play important roles during evolution in potato. The detailed information of syntenic gene pairs is provided in Supplementary Table S3.

### 3.5. Chromosomal Distribution and Duplication Events

**Figure 4:**
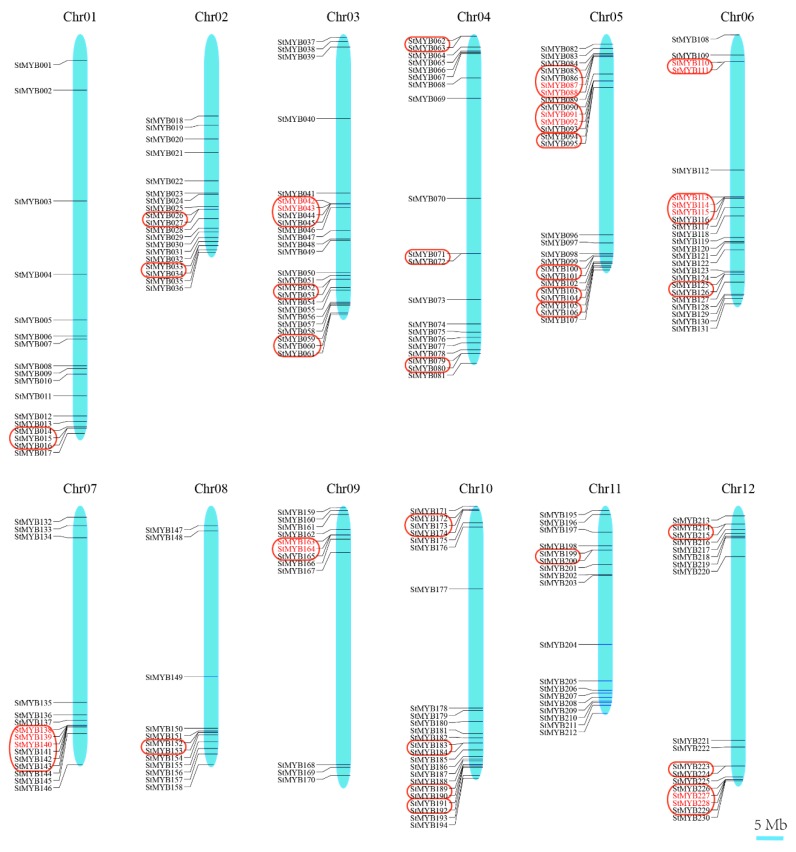
Distribution of *StMYB* genes on potato chromosomes. In potato, 230 *StMYB* genes were successfully mapped to 12 potato chromosomes. The red box indicated the gene cluster, while the tandem duplication pair was featured by red color.

The 230 *StMYB* genes mapped on potato chromosomes were unevenly distributed on the 12 chromosomes (Figure 4). The chromosome 5 harbored the most (26) and the chromosomes 08 and 09 harbored the least number (12) of potato *MYB* family genes. As defined in an earlier study [48], adjacent *StMYB* genes located within 200 kb were defined as forming a cluster, and *StMYB* genes sharing more than 70% identity in a cluster were considered as tandem duplication genes. A total of 76 *StMYB* genes were thereby identified to form 29 clusters on different chromosomes. In addition, eight tandem duplication arrays consisting of 18 *StMYB* genes were identified. 

**Figure 5:**
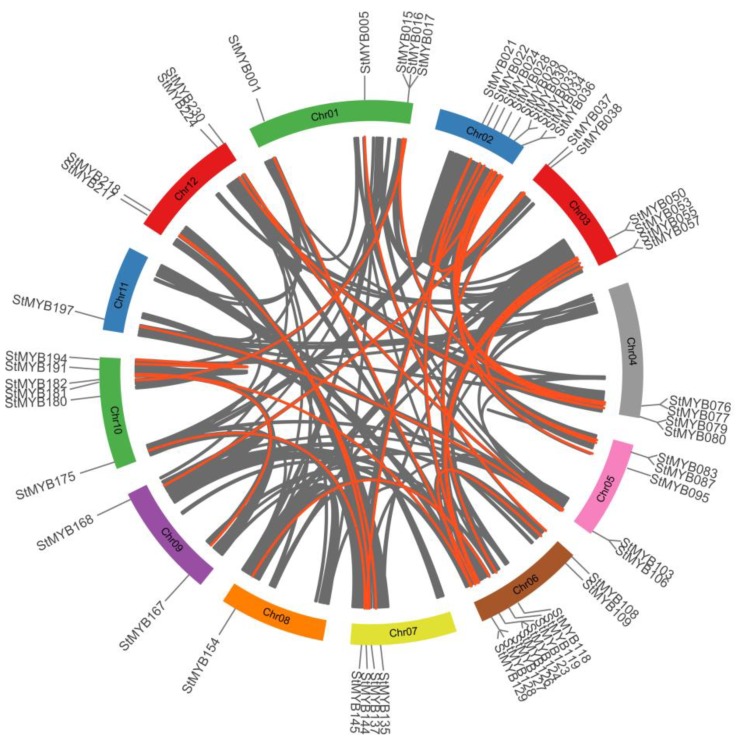
Segmental duplication events and inter-chromosomal relationships between *StMYB* genes. The 31 putative segmental duplication pairs of *StMYB* genes were investigated with MCScanX and linked by the colored lines, respectively. The gray lines indicate all putative segmental duplication pairs in the potato genome.

Furthermore, segmental duplication or whole genome duplication analysis of the *StMYB* genes was performed using the MCScanX. Totally, 31 segmental duplication pairs with 57 *StMYB* genes were identified (Figure 5). These results suggested that about 32.2% of the *StMYB* genes may be generated by duplication events, which played the major role in the expansion of the *MYB* gene family in potato. All of the tandem and segmental duplication genes were listed in Supplementary Table S4.

The Ka/Ks ratio is used to estimate the balance between neutral mutations, purifying selection, and beneficial mutations. The Ka/Ks ratios of the tandem and segmental duplication gene pairs were calculated. As a result, all of the Ka/Ks ratios were less than 1, suggesting that the potato *MYB* duplicated members may have experienced purifying selective pressure during evolution.

### 3.6. Promoter Analysis of StMYB Genes

**Figure 6:**
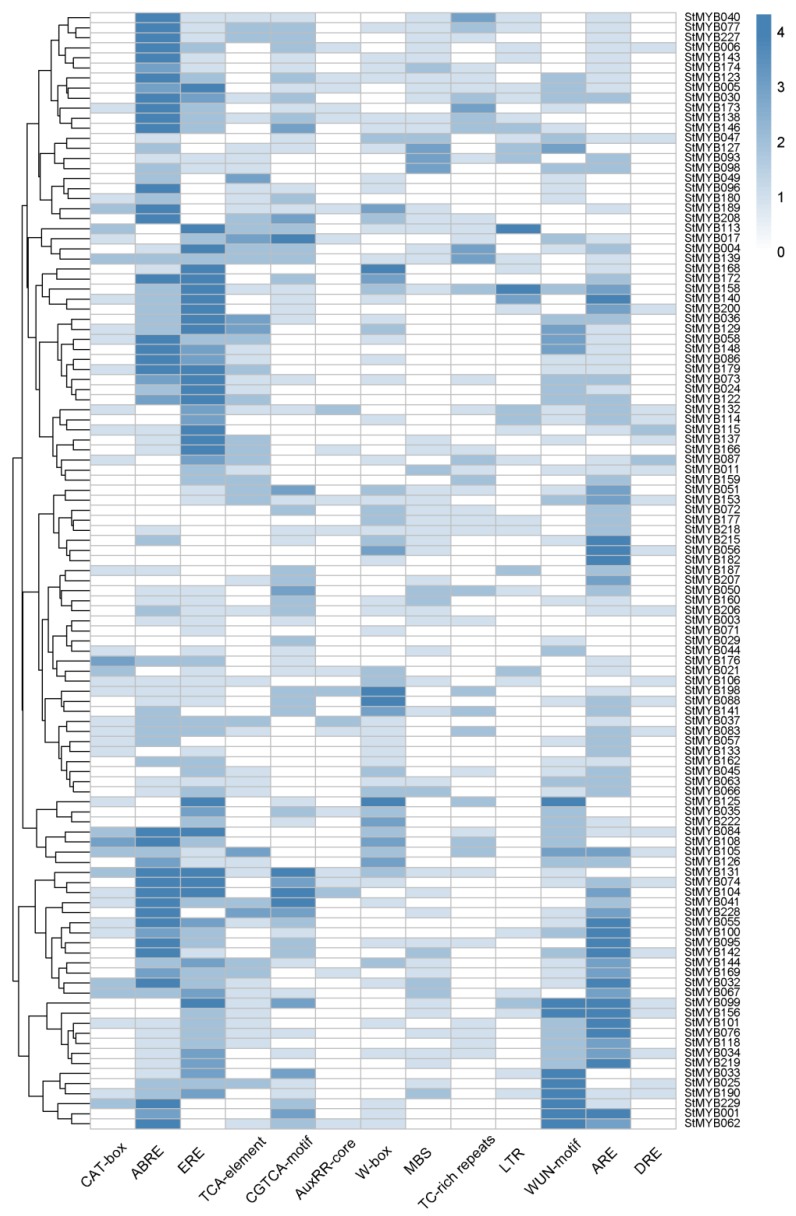
Regulatory elements in the promoter regions of *StMYB* genes.

To study the expression regulation of the *StMYB* genes, putative cis-elements on promoter regions were predicted using PlantCARE (Figure 6). Cis-elements related to developmental processes, such as meristem development (CAT-box), were detected in promoter regions of certain *StMYB* genes. The hormone-responsive elements were also identified in the promoters, including ABRE, ERE, TCA-element, CGTCA-motif, and AuxRR-core, which mediate the plant responses to ABA, ethylene, salicylic acid (SA), methyl jasmonate (MeJA), and auxin, respectively. Interestingly, the ABRE element was detected in promoter regions of most of the *StMYB* genes, implying those *StMYB* genes might participate in ABA-mediated stress responses. Furthermore, stress-responsive elements including WRKY binding site (W-box), MYB binding site (MBS), stress-responsive element (TC-rich repeats), heat stress-responsive element (HSE), low-temperature-responsive element (LTR), wound-responsive element (WUN-motif), and anaerobic induction element (ARE) were observed to be abundant in the promoters of a large number of *StMYB* genes, suggested that these genes may participate in the responses to various stress conditions.

### 3.7. Expression of StMYB Genes in Different Tissue Types

**Figure 7:**
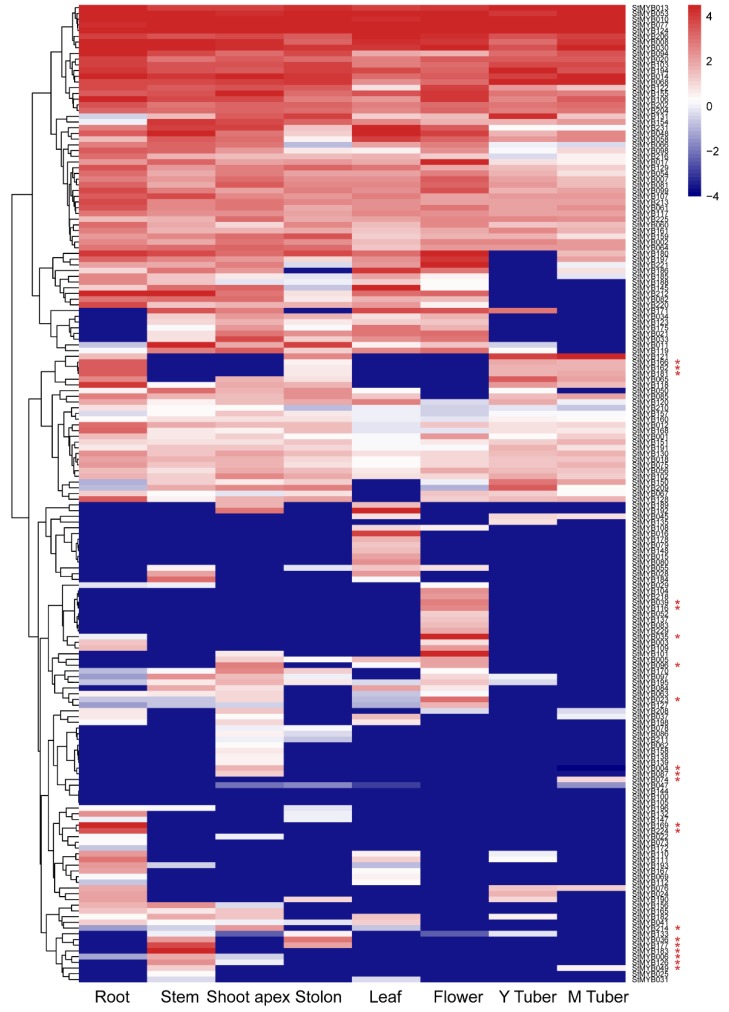
The expression patterns of the *StMYB* genes in the tested tissues. The expression pattern data were retrieved from transcriptome data and visualized by R Programming Language. Y Tuber, young tuber; M Tuber, mature tuber.

The gene expression patterns could provide information for potential gene function. The RNA-Seq data for *StMYB* genes in eight representative tissues, including root, stem shoot apex, stolon, leaf, flower, young tuber, and mature tuber, were obtained from the PGSC and analyzed [51]. The expression data for 171 *StMYB* genes were detected in at least one of the eight tested tissues, with a large number of *StMYB* genes being highly expressed in all eight tissues (Figure 7). While, a number of *StMYB* genes exhibited tissue-specific expression pattern either in only one or more tissues. For example, *StMYB169* and *StMYB224* were expressed exclusively in roots. The expression of *StMYB006*, *StMYB049*, *StMYB126*, and *StMYB183* was detected in the stem only. Other tissue-specific expression patterns were also detected, including *StMYB004*, *StMYB08.7* and *StMYB214* in the shoot apex, *StMYB035*, *StMYB039*, and *StMYB116* in flowers, as well as *StMYB074* in mature tubers. In addition, *StMYB036* and *StMYB177* were highly expressed in stem/stolon, *StMYB023* and *StMYB096* were highly expressed in shoot apex/flower, while high-level expression of *StMYB162*, *StMYB166*, and *StMYB181* was detected in root, young, and mature tuber tissues.

### 3.8. Expression Patterns of StMYB Genes in Response to Stress Treatments

**Figure 8:**
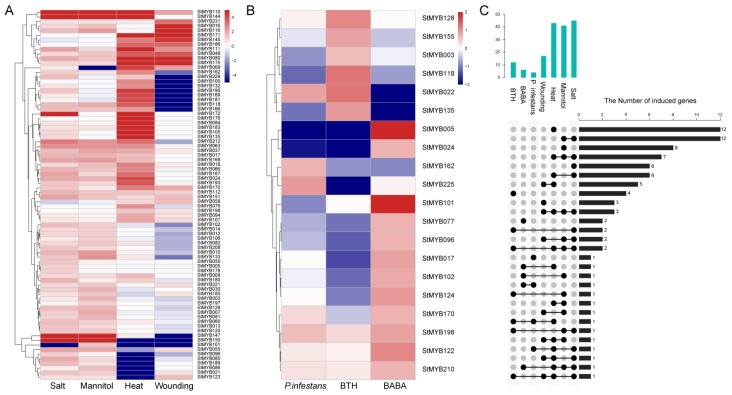
The expression patterns of *StMYB* genes under abiotic and biotic stress treatments. (**A**) The relative expression ratios of abiotic stress treatments; (**B**) the relative expression ratios of biotic stress treatments; (**C**) the summarized information of the stress-induced *StMYB* genes. The relative expression ratios of abiotic and biotic stress treatments were calculated relative to the untreated control, respectively, and then the significantly induced gene was defined to possess a log2 relative expression ratio ≥1 under one of the stress treatments. The red color, white color, and blue color represent the up-regulated, unaltered, and down-regulated expression, respectively.

To investigate the stress-responsiveness *StMYB* genes, the RNA-Seq data for different abiotic stress treatments including salinity, drought, heat, and wounding were analyzed (Figure 8). As a result, a total of 80 *StMYB* genes were significantly induced under one or more of these treatments (Figure 8A), among which, *StMYB080*, *StMYB110*, and *StMYB112* were highly expressed under all the four stress treatments, whereas, *StMYB037*, *StMYB063*, *StMYB066*, *StMYB144*, *StMYB166*, *StMYB168*, and *StMYB212* were induced by all the stress conditions except wounding. Furthermore, *StMYB048* and *StMYB111* showed higher expression in responses to heat and wounding. Further, *StMYB069*, *StMYB145*, *StMYB171*, *StMYB175*, and *StMYB186* were induced only after heat and wounding treatments while *StMYB030*, *StMYB110*, *StMYB144*, *StMYB147*, and *StMYB150* were highly expressed in response to salt and drought stresses.

The expression changes of *StMYB* genes in response to biotic stresses were also analyzed. RNA-seq data from *Phytophthora infestans* inoculation and application of two chemical elicitors, BABA and BTH were used for analysis (Figure 8B). The results showed that none of the *StMYB* gene was explicitly expressed after treatments of all three biotic stresses. A total of 20 *StMYB* genes were induced by one or more of the biotic stress conditions. *StMYB022* was found to be up-regulated under both BABA treatment and *P. infestans* inoculation. *StMYB225* was only induced by *P. infestans* inoculation. Expression of *StMYB128* and *StMYB155* was induced by BABA treatment only, whereas *StMYB077*, *StMYB122*, *StMYB124*, and *StMYB210* were induced only by BTH treatment. 

Moreover, a number of *StMYB* genes were induced under both abiotic and biotic stress conditions (Figure 8C). For example, *StMYB118* was found to be induced by BABA and under all the tested abiotic stress treatments except wounding. *StMYB017*, *StMYB024*, and *StMYB170* were up-regulated by BTH and in response to salt and heat treatments. Similarly, *StMYB162* was highly induced by *P. infestans* inoculation and under heat and salt stress treatments. The numbers of *StMYB* genes that were significantly induced by specific stress treatment are summarized in Supplementary Table S5, including 8 genes induced by drought, 12 induced by heat, 3 induced by wounding, 1 induced by *P. infestans*, 2 induced by BABA, and 3 induced by BTH treatment, respectively (Figure 8C and Supplementary Table S5).

### 3.9. Validation of Expression Patterns by qRT-PCR

**Figure 9:**
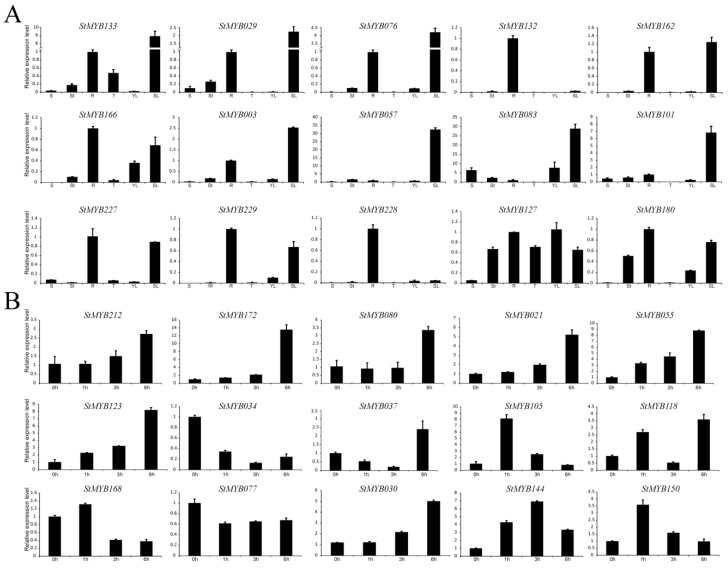
The expression patterns of selected *StMYB* genes detected by qRT-PCR. (**A**) To confirm the tissue specificity, the expression pattern of the selected *StMYB* genes was calculated as folds relative to the expression level of the root. (**B**) The expression pattern of selected *StMYB* genes in response to salt stress treatments, which was calculated as folds relative to the untreated control.

To confirm the expression changes of the *StMYB* genes from RNA-seq analysis, representative *StMYB* genes were selected to perform qRT-PCR analysis (Figure 9). Overall, the qRT-PCR data of representative genes agreed with the results of the RNA-seq analysis. A minor difference exists potentially due to variation caused by samples collecting methods and difference in developmental status.

As results, *StMYB133* was found to express in the root, shoot tip, tuber, and senescent leaf specifically (Figure 9A). In subgroup 14, *StMYB029*, *StMYB076*, *StMYB132*, *StMYB162*, and *StMYB166* were found to share similar expression pattern and highly expressed in the root, indicted these homologs may act redundantly. In subgroup 20, *StMYB003*, *StMYB057*, *StMYB083*, *StMYB101*, *StMYB227*, and *StMYB229* were observed to highly expressed in senescent leaf. Notably, *StMYB228* were also grouped with *AtMYB2* in subgroup 20, which did not show abundant transcripts in senescent leaf.

Furthermore, inconsistent with the results from transcriptome data, several *StMYB* genes were significantly induced by salt treatments, such as *StMYB212, StMYB172*, and *StMYB080* (Figure 9B). In subgroup 1, *StMYB021*, *StMYB055*, and *StMYB123* significantly responded to salt treatments, whereas *StMYB034* were repressed by salt. Furthermore, in subgroup 2, *StMYB037*, *StMYB105*, *StMYB118*, and *StMYB168* responded to the salt treatment in a different manner. Interestingly, in subgroup 22, *StMYB077* was down-regulated by salt stress treatment, whereas *StMYB030* was significantly induced by salt treatment.

### 3.10. Subcellular Localization Analysis

**Figure 10:**
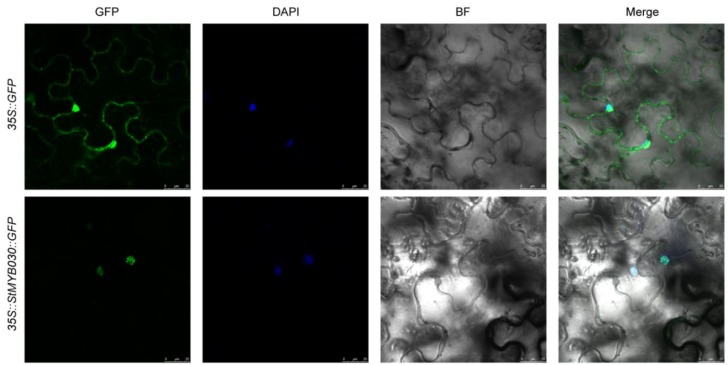
The subcellular localization of StMYB030 in tobacco epidermal cells. The *StMYB030-GFP* fusion construct and *GFP* gene driven by the CaMV-35S promoter were transiently expressed into tobacco, respectively. DAPI (dye 4,6-diamidino-2-phenylindole) staining indicted the nucleus.

To further explore the potential function of the *StMYB* genes, the subcellular localization of the one of the stress-responsive genes, *StMYB030*, was analyzed (Figure 10). The coding sequence of *StMYB030* without the stop codon was fused with the *GFP* reporter gene, which was driven by the CaMV35S promoter. The *Agrobacterium* cultures with the recombinant construct and the 35S::GFP control were used to inject the tobacco leaf epidermal cells. As revealed by confocal microscopy, the green fluorescence of the StMYB030-GFP fusion protein was specifically confined within the nucleus, which was further verified by DAPI staining, whereas the signal of GFP protein was found to distribute throughout the whole cell.

### 3.11. Overexpression of StMYB030 Gene Enhanced Salt Tolerance in Arabidopsis

**Figure 11:**
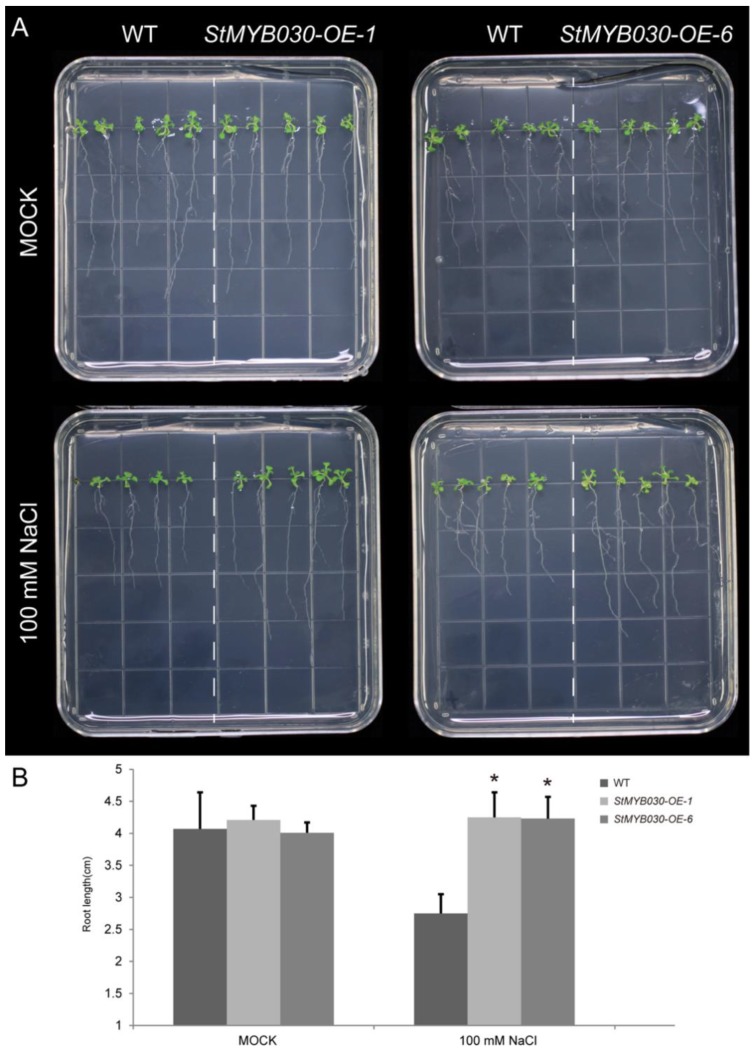
The Effects of salt stress treatment on root growth of *StMYB030* gene overexpressing lines in transgenic *Arabidopsis*. (**A**) The primary root length of wildtype and *StMYB030* gene overexpression lines under salt treatments in transgenic *Arabidopsis*. (**B**) The quantification of primary root length under normal condition and 100 mM NaCl treatments. The data were retrieved from three biological replicates. WT, wildtype. The data were means ± SD of three biological repeats. * *p* < 0.05 (*t*-tests).

The function of *StMYB030* gene was further examined via ectopic expression in *Arabidopsis*. The root elongation assay was performed to examine the salt tolerance of wildtype and *StMYB030* overexpressing *Arabidopsis* (Figure 11 and Supplementary Figure S3). There was no significant difference in root length between wildtype and the *StMYB030* overexpressing plants under normal conditions. However, compared to the wildtype, significantly longer roots of the transgenic plants were observed after 14 days growing on 100 mM NaCl plates. Then, two independent overexpression lines displayed the similar phenotype with longer roots under salt stress treatments. Taken together, these results indicated that the overexpression of *StMYB030* gene improved the salt tolerance in transgenic *Arabidopsis*.

## 4. Discussion

The MYB transcription factors have been reported to be one of the largest gene families in plants and play important roles in plant development, metabolism, and various stress responses [3]. In the current study, BLASTP searches were performed to identify the MYB members in potato. The identified potato *MYB* genes were studied via analyses of phylogeny, gene structure and motif organization, syntenic analysis, chromosomal distribution, duplication event, and expression profiles. Moreover, the homologous counterparts between *Arabidopsis* and potato *R2R3-MYB* genes were investigated to predict their potential functions.

A total of 233 *MYB* genes were identified in the potato genome, including 119 R1-MYB, 112 R2R3-MYB, and 2 R1R2R3 MYB members. Segmental and tandem duplication events were found to play important roles in the expansion of the MYB family in potato. A total of 31 segmental duplication pairs and 10 tandem duplication pairs were identified, with all of these duplicated pairs sharing the Ka/Ks ratios <1, suggesting that these duplicated *StMYB* genes might have undergone purifying selection and might have maintained conserved functions during evolution. In some occasions, different cis-elements were detected on promoter regions of the duplicated gene pairs such as *StMYB030/StMYB077*, implying the diversity of potential functions.

As the major MYB subgroup in plant, R2R3-MYB members have evolved from the R1R2R3-MYB ancestors by losing their R1 repeat [5]. R2R3-MYB members are featured by two MYB repeats which function in the DNA binding. The sequence features of the R2 and R3 repeat were highly conserved between potato and *Arabidopsis*. The similar gene structure and motif organization were observed in R2R3-MYB members within the same subgroup, supporting the results of phylogenetic analysis. The subgroup 29 only harbored R2R3-MYB members from potato, implying the member of this subgroup may have arisen after the divergence of potato and *Arabidopsis*. Interestingly, except *StMYB219* and *StMYB233*, all genes from subgroup 29 were found in five clusters, indicating tandem duplication events may have played a significant role in the expansion of this subgroup during evolution.

A number of R2R3-MYB members have been characterized to participate in regulating plant development. In subgroup18, AtMYB33 and AtMYB65 have been reported to facilitate anther and pollen development [10]. StMYB001, StMYB127, StMYB137, and StMYB218 were clustered together with AtMYB33/AtMYB65 in subgroup 18 (Figure 2) and all of these four MYB encoding genes showed abundant expression in flowers (Figure 7), suggesting possible involvement of these homologous members in flower development. Similarly, *AtMYB68* in subgroup 14 was reported to specifically regulate root growth [12], and its potato homologs *StMYB076* and *StMYB132* were found to be highly expressed in the root (Figure 7 and Figure 9A). In subgroup 15, *AtMYB0* and *AtMYB23* were reported to function in the origination of trichome in shoots [13,14], whereas their potato homolog *StMYB133* (Figure 7 and Figure 9A) was found to be expressed in stem, stolon, and tuber. All these data suggested functional conservation between homologous R2R3-MYB members from *Arabidopsis* and potato.

In *Arabidopsis*, AtMYB11/PFG2, AtMYB12/PFG1, and AtMYB111/PFG3 in subgroup 7 have been reported to control flavonol biosynthesis in all tissues [17], and StMYB005, StMYB104, and StMYB108 were close homologs of these PFG members (Figure 2). In addition, *StMYB005* and *StMYB108* were predicted to form syntenic gene pairs with *AtMYB11* (Figure 3 and Supplementary Table S3). *StMYB005/StMYB108* genes were identified as a segmental duplication pair (Figure 5). However, *StMYB005*, *StMYB104*, and *StMYB108* exhibited different expression patterns. *StMYB005* was found to be expressed in stem and leaf, whereas *StMYB108* was expressed in flower and leaf (Figure 7), indicating possible functional divergence between these R2R3-MYB homologs.

*AtMYB2* has been reported to be up-regulated in senescent leaves and involved in regulation of leaf senescence [16]. AtMYB108 was found to bind to specific regions of the *ANAC003* promoter to form a MYB-NAC regulatory network, which participated in leaf senescence regulation [56]. In this study, the phylogenetic analysis revealed StMYB101, StMYB003, StMYB227, StMYB228, StMYB229, StMYB057, and StMYB083 were clustered together with AtMYB2 and AtMYB108 in subgroup 20 (Figure 2). The qRT-PCR results indicated that these potato *MYB* genes, except for *StMYB228*, were highly expressed in senescent leaves (Figure 9), suggesting that these homologous genes might also participate in the regulation of leaf senescence. Interestingly, although *StMYB227* and *StMYB228* were predicted to have arisen from tandem duplication events (Figure 4), *StMYB227* was highly expressed in senescent leaves but *StMYB228* was not (Figure 9), indicating functional divergence between these duplicated genes in regulating leaf senescence. Notably, *StMYB003* and *StMYB229* were found to be induced by drought stress and *StMYB101* was responsive to heat (Figure 8), suggesting that these genes may function in coordinating leaf senescence and stress responses in potato.

A large number of R2R3-MYB family members were found to confer tolerance to abiotic and biotic stresses in plants [2]. In subgroup1, AtMYB30, AtMYB60, and AtMYB96, which have been reported to be involved in abiotic stress responses [24,25,27], were clustered together with StMYB021, StMYB034, StMYB055, StMYB123, and StMYB189 (Figure 2). In agreement with their *Arabidopsis* homologs, the potato genes *StMYB021*, *StMYB055*, and *StMYB123* were found to be induced by salt treatments and *StMYB189* was induced by heat/wounding (Figure 8 and Figure 9B). *StMYB021/StMYB034* was a segmental duplication pair but unlike *StMYB021*, *StMYB034* was not responsive to any of the testes stress treatments (Figure 5). Functional divergence might have occurred within this pair. In subgroup 2, AtMYB15 was reported to mediate environmental signals and stresses tolerance [30,31] and potato genes *StMYB037*, *StMYB105*, *StMYB118*, and *StMYB168* in the same subgroup were induced by at least one stress treatment, implying that the potato R2R3-MYB members in this subgroup may also confer stress tolerance (Figure 8).

Also, in subgroup22, StMYB071, StMYB072, StMYB030, and StMYB077 were clustered together with *Arabidopsis* members AtMYB44, AtMYB70, AtMYB73, and AtMYB77 (Figure 2), which have been reported to function in regulating ABA-mediated stomatal closure and abiotic stress responses [29]. In addition, *StMYB030/StMYB077* formed syntenic gene pairs with *AtMYB44* (Figure 3 and Supplementary Table S3). Both *StMYB030* and *StMYB077* possessed ABRE elements in their promoters (Figure 6), suggesting that they might be involved in ABA signaling and stress response. Furthermore, *StMYB030* was significantly induced by salt and drought treatments (Figure 8 and Figure 9B) and the StMYB030-GFP fusion protein was found to be localized in the nucleus, suggesting that StMYB030 may act as a transcription factor regulating stress responses (Figure 10). Indeed, the overexpression analysis indicated that StMYB030 could confer salt tolerance in transgenic *Arabidopsis* (Figure 11).

## 5. Conclusions

In summary, a systematic investigation was performed to identify and characterize the MYB family members in potato. The characterization of the potato MYB family members provided insights into the evolutionary relationship between MYB family members. Notably, the homologous counterparts between *Arabidopsis* and potato MYB members were found to likely play conserved roles in regulating plant development and stress responses. Notably, the *StMYB030* gene was found to be induced by salt and drought stress treatments and able to confer salt tolerance in transgenic *Arabidopsis*. The results provided here could be helpful in the future exploration of the biological functions of these potato *MYB* genes.

## Supplementary Materials

The following are available online at https://www.mdpi.com/2218-273X/9/8/317/s1, Supplementary Table S1. The qRT-PCR primers used in this study; Supplementary Table S2. The detail information of identified potato MYB family members; Supplementary Table S3. The syntenic pairs between potato and other five plant species; Supplementary Table S4. The detail information of segmental and tandem duplication gene pairs; Supplementary Table S5. The detail information of the induced *StMYB* gene under various stress treatments; Supplementary Figure S1. The motif and gene structure organizations of StMYB members; Supplementary Figure S2. The putative conserved motifs in StMYB proteins; Supplementary Figure S3. The expression level of *StMYB030* gene in wildtype and two overexpression lines, the ratios of gene expression level were calculated relative to the wildtype.
