# Supplementary material for: Systematic Analysis of MYB Family Genes in Potato and Their Multiple Roles in Development and Stress Responses
Source: Biomolecules. 2019 Jul 30;9(8):317. doi: 10.3390/biom9080317 (PMC6723670; doi:10.3390/biom9080317)
Supplement: Supplementary file 1 [file biomolecules-09-00317-s001.pdf]

Supplymentary

Table S1. Primers in this study.

Gene name	Forward Primer-Sequence (5'-3')	Reverse Primer- Sequence (5'-3')
StMYB003	TACAATTATCCCCAACAACAAC	GATCCATATCTGCAGCAGCAGC
StMYB021	CATTTGAGTCACTATTTGGTTT	CATGTTCATTTTCTTCCTTCTT
StMYB029	TCCTCCATCAGTATTCGAAACC	GAATAGGCACCACACTGAAAAG
StMYB030	TTGGCTGAGTTCTCTAATCGTT	CAAATACCTTGTCTGGCTGTTC
StMYB034	AATCAAGCCCAATTCCTGACTA	GCCTTTGATTGCTCGGAATTAT
StMYB037	TGATCGGTCATGTCATATAGGC	AAGCCTTGCATTCTACGGATAA
StMYB055	CTTAGTAGCTAGCCTGAGCAAA	GCACATAGTACAAACAGTTGAAGC
StMYB057	TTACTCCACGCCAGATAGATGA	TTTCCAGTTGCTAATTGTCCAA
StMYB076	CCATCATGCAACAACCAATACA	TCATGTTGTTGAAGACGTTGTC
StMYB077	AGTGAAGAGTTACATGTCTGGG	TCCATTGTTTCTTCTCCAGTGT
StMYB080	TATGCCAATGAACATAGGTATC	GATTAGTTTTGATTTGTGATAC
StMYB083	ACAACAAATCCCATGATACTAA	CACCTCACATTCTGAAATTGAC
StMYB101	CGACGTGGCAATATTACTCTTG	AGCGATTTTAGACCAACGATTG
StMYB105	CAAACAAGGAAGAGAACATGCA	TTCGCCTCTAACTGATCTCATC
StMYB108	AACTTGATTGTTGGGATTGGGA	TTTTCTGGTGTTGCTTCTTTGT
StMYB123	GGTTTCAAGTTCTAGCCCTAGT	GGATTGCGAAACATCCGAATTA
StMYB127	GCTCGAGCTCCCTTCACTCCAA	GTCCAACAGACCGCTGTTCCTA
StMYB132	GGCAAAAAGTTGGGATCAATGA	ATCAATCTCCAGCTGAGTGAAA
StMYB133	GGGAAACAGAGTAATCTCAGGT	TCTTTTCGGAAGCAATTGTCAC
StMYB144	GGCAACAAATGGTCAGCTATAG	GCCTTTTTCTAATGTGGGTGTT
StMYB150	ACAGCAGATCAAAACAGAGAGA	GTGCCTCTATTCTCATTTGCAG
StMYB162	CTATTGGAACACGAAGCTCAAG	AGATGGAGCTTGAAGAAGTGAA
StMYB166	CTAGTACATTCACGGGTCCTAC	CATCAACATGACAACCACTACC
StMYB168	TGATTCCAAAGCTCCTTGTACT	AGTCCATTTGATCACTACCGTT
StMYB172	CTAAGAATGCTGGGCTTCAAAG	GCAAGTGGATGATTGTCTCTTC
StMYB180	CTCCACATCATCAACAACATCG	CATTAGTCTTCAAGCCCAAAAA
StMYB212	AGTTGAAGGAGAAAAGAAATCT	TCCAAAGTGAGTCTAGGGAATA
StMYB227	CGAGTCCAAAAACATGCAAAAC	AGTTTTCTGATGAAGTTGCCAC
StMYB228	GGGCAATAGGTGGTCAAAAATT	GGGTTGTTGAGTATCGCTACTA
StMYB229	CTCGTTCTGCAGGTTTAAAGAG	GTGCAATTTTCGACCACCTATT

Table S2. The related information of the MYB genes of potato.

Chromosome No.	Gene Name	Gene ID	ORF length（bp）	Size（aa）	MW(Da)	pI	Group
1	StMYB001	PGSC0003DMT400058426	1572	523	57332.39	5.15	S18
1	StMYB003	PGSC0003DMT400022600	894	297	34370.2	6.59	S20
1	StMYB004	PGSC0003DMT400062437	1017	338	38050.91	9.06	S9
1	StMYB005	PGSC0003DMT400023322	1092	363	40888	5.03	S7
1	StMYB006	PGSC0003DMT400017709	1068	355	39534.87	5.71	S16
1	StMYB011	PGSC0003DMT400047076	1281	426	47675.73	6.92	S13
1	StMYB017	PGSC0003DMT400015803	765	254	28785.57	8.76	S4
2	StMYB021	PGSC0003DMT400017844	993	330	36728.92	5.38	S1
2	StMYB024	PGSC0003DMT400057690	1113	370	42268.92	5.99	S11
2	StMYB025	PGSC0003DMT400018897	963	320	36426.04	6.42	S30
2	StMYB029	PGSC0003DMT400055148	993	330	37677.05	7.12	S14
2	StMYB030	PGSC0003DMT400064306	1116	371	41161.19	8.65	S22
2	StMYB032	PGSC0003DMT400003479	717	238	27720.23	6.97	S31
2	StMYB033	PGSC0003DMT400003382	1254	417	45431.21	5.68	S9
2	StMYB034	PGSC0003DMT400003347	957	318	36181.45	6.54	S1
2	StMYB035	PGSC0003DMT400001278	624	207	24004.5	8.59	S19
2	StMYB036	PGSC0003DMT400032930	759	252	29291.97	5.59	S26
3	StMYB037	PGSC0003DMT400034878	759	252	29248.75	5.09	S2
3	StMYB040	PGSC0003DMT400022009	882	293	33241.33	6.79	S27
3	StMYB041	PGSC0003DMT400053433	837	278	31939.74	8.32	S34
3	StMYB044	PGSC0003DMT400093355	726	241	27970.03	8.66	S29
3	StMYB045	PGSC0003DMT400021498	723	240	27490.22	8.46	S29
3	StMYB047	PGSC0003DMT400037080	837	278	31568.15	6.31	S16
3	StMYB049	PGSC0003DMT400079714	735	244	28864.12	8.99	S21
3	StMYB050	PGSC0003DMT400046645	978	325	36999.18	5.64	S13
3	StMYB051	PGSC0003DMT400046600	1065	354	41526.24	5.28	S25
3	StMYB055	PGSC0003DMT400050287	1014	337	37205.31	5.68	S1
3	StMYB056	PGSC0003DMT400014418	2142	713	79926.32	9.07	S33
3	StMYB057	PGSC0003DMT400014383	837	278	31878.49	6.6	S20
3	StMYB058	PGSC0003DMT400014350	894	297	32639.4	6.59	S34
4	StMYB062	PGSC0003DMT400007336	984	327	37798.19	6.22	S9
4	StMYB063	PGSC0003DMT400007351	1200	399	45477.93	6.29	S25
4	StMYB066	PGSC0003DMT400068798	1413	470	53274.4	6.71	S25
4	StMYB067	PGSC0003DMT400065102	804	267	29503.79	6.53	S34
4	StMYB071	PGSC0003DMT400085006	603	200	23173.66	9.9	S22
4	StMYB072	PGSC0003DMT400095287	915	304	34252.29	9.3	S22
4	StMYB073	PGSC0003DMT400063862	909	302	34959.47	9.51	S21
4	StMYB074	PGSC0003DMT400016394	1062	353	40021.15	6.03	S24
4	StMYB076	PGSC0003DMT400012702	990	329	37247.96	7.58	S14

4	StMYB077	PGSC0003DMT400008569	963	320	35020.04	9.24	S22
5	StMYB083	PGSC0003DMT400037716	822	273	31951.82	6.16	S20
5	StMYB084	PGSC0003DMT400078477	537	178	20710.65	9.14	S4
5	StMYB086	PGSC0003DMT400047415	1290	429	47643.66	7.63	S21
5	StMYB087	PGSC0003DMT400047379	999	332	37891.7	8.56	S9
5	StMYB088	PGSC0003DMT400047446	1005	334	38145.13	8.72	S9
5	StMYB093	PGSC0003DMT400091420	696	231	26183.99	8.82	S34
5	StMYB095	PGSC0003DMT400031250	654	217	25464.97	6.39	S26
5	StMYB096	PGSC0003DMT400055763	960	319	35708.74	5.97	S9
5	StMYB098	PGSC0003DMT400069753	747	248	28943.59	8.84	S34
5	StMYB099	PGSC0003DMT400069921	1125	374	40664.72	6.28	S23
5	StMYB100	PGSC0003DMT400069955	690	229	27300.93	8.81	S31
5	StMYB101	PGSC0003DMT400069841	975	324	37488.49	6.13	S20
5	StMYB104	PGSC0003DMT400060236	1401	466	51865.37	4.59	S7
5	StMYB105	PGSC0003DMT400060168	663	220	25823.76	6.25	S2
5	StMYB106	PGSC0003DMT400060403	567	188	21509.83	5.46	S34
6	StMYB108	PGSC0003DMT400018841	912	303	35039.87	4.72	S7
6	StMYB113	PGSC0003DMT400070912	783	260	29823.53	6.97	S29
6	StMYB114	PGSC0003DMT400051513	744	247	28566.37	6	S29
6	StMYB115	PGSC0003DMT400051510	732	243	28106.53	5.92	S29
6	StMYB118	PGSC0003DMT400042007	807	268	30766.49	6.2	S2
6	StMYB122	PGSC0003DMT400080427	858	285	31845.26	9.09	S4
6	StMYB123	PGSC0003DMT400083076	1128	375	41558.81	5.83	S1
6	StMYB125	PGSC0003DMT400087324	1293	430	49637.43	5.81	S25
6	StMYB126	PGSC0003DMT400069644	948	315	36246.5	6.9	S13
6	StMYB127	PGSC0003DMT400015156	1509	502	54999.22	5.5	S18
6	StMYB129	PGSC0003DMT400078029	924	307	34764.94	9	S34
6	StMYB131	PGSC0003DMT400051682	777	258	29736.92	4.88	S2
7	StMYB132	PGSC0003DMT400048261	1107	368	41807.79	6.34	S14
7	StMYB133	PGSC0003DMT400079416	648	215	25001.7	8.97	S15
7	StMYB137	PGSC0003DMT400074486	1455	484	53619.52	6.2	S18
7	StMYB138	PGSC0003DMT400067321	738	245	28426.7	6.12	S29
7	StMYB139	PGSC0003DMT400067322	738	245	28536.45	5.45	S29
7	StMYB140	PGSC0003DMT400067318	732	243	28219.27	5.78	S29
7	StMYB141	PGSC0003DMT400097604	633	210	24490.44	7.01	S29
7	StMYB142	PGSC0003DMT400039029	732	243	28243.17	6.8	S29
7	StMYB143	PGSC0003DMT400086165	864	287	33518.49	6.76	S29
7	StMYB144	PGSC0003DMT400047219	1002	333	38101.68	5.63	S11
7	StMYB146	PGSC0003DMT400089342	726	241	27609.17	6.83	S3
8	StMYB148	PGSC0003DMT400014983	972	323	36715.45	9.29	S28
8	StMYB153	PGSC0003DMT400045182	972	323	36629.89	6.39	S11
8	StMYB156	PGSC0003DMT400012203	846	281	31790.8	5.85	S26

8	StMYB158	PGSC0003DMT400031607	822	273	31515.28	9.21	S28
9	StMYB159	PGSC0003DMT400052411	867	288	32620.47	8.36	S34
9	StMYB160	PGSC0003DMT400052395	1140	379	41043.35	5.38	S23
9	StMYB162	PGSC0003DMT400009926	903	300	33622.52	6.65	S14
9	StMYB166	PGSC0003DMT400005085	903	300	33555.44	6.78	S14
9	StMYB168	PGSC0003DMT400044362	807	268	30238.84	5.45	S2
9	StMYB169	PGSC0003DMT400003796	762	253	29824.52	9.49	S31
10	StMYB172	PGSC0003DMT400029257	942	313	35680.17	7.04	S11
10	StMYB173	PGSC0003DMT400029235	720	239	27675.86	7.14	S3
10	StMYB174	PGSC0003DMT400022402	987	328	36882.2	7.65	S27
10	StMYB176	PGSC0003DMT400068481	1008	335	38445.92	6.37	S11
10	StMYB177	PGSC0003DMT400040774	1224	407	46054.28	7.19	S13
10	StMYB179	PGSC0003DMT400085829	558	185	21547.73	9.99	S28
10	StMYB180	PGSC0003DMT400034372	807	268	30567.53	8.49	S4
10	StMYB182	PGSC0003DMT400049209	858	285	32779.67	7.17	S34
10	StMYB187	PGSC0003DMT400072687	411	136	16104.65	9.94	S32
10	StMYB189	PGSC0003DMT400072272	819	272	31019.69	6.21	S1
10	StMYB190	PGSC0003DMT400072258	966	321	36801.07	6.02	S21
11	StMYB198	PGSC0003DMT400023903	891	296	32252.28	4.23	S34
11	StMYB200	PGSC0003DMT400071438	651	216	24475.59	9.37	S34
11	StMYB206	PGSC0003DMT400019099	975	324	35145.1	6.31	S33
11	StMYB207	PGSC0003DMT400020979	600	199	22246.57	5.12	S33
11	StMYB208	PGSC0003DMT400020790	1716	571	64652.56	5.78	S34
12	StMYB215	PGSC0003DMT400000932	870	289	33065.57	5.45	S14
12	StMYB218	PGSC0003DMT400036086	1431	476	53068.46	6.44	S18
12	StMYB219	PGSC0003DMT400035748	696	231	26723.2	6.25	S29
12	StMYB222	PGSC0003DMT400011167	813	270	31477.32	5.88	S7
12	StMYB227	PGSC0003DMT400011745	705	234	27770.94	6.6	S20
12	StMYB228	PGSC0003DMT400011750	693	230	27296.31	6.08	S20
12	StMYB229	PGSC0003DMT400011748	888	295	34472.98	5.87	S20
0	StMYB233	PGSC0003DMT400097096	642	213	25029.64	6.14	S29

Table S**3.** One-to-one orthologous relationships between potato and other five plant species.

Common collinear StMYB gene pairs in rice and maize	Common collinear StMYB gene pairs in grape, tomato and Arabidopsis	Common collinear StMYB gene pairs in grape, tomato, Arabidopsis, rice and maize
StMYB005	StMYB003	StMYB005
StMYB011	StMYB005	StMYB011
StMYB017	StMYB007	StMYB017
StMYB028	StMYB008	StMYB028
StMYB059	StMYB009	StMYB059
StMYB060	StMYB011	StMYB060
StMYB129	StMYB013	StMYB129
StMYB159	StMYB014	StMYB159
StMYB161	StMYB015	StMYB161
StMYB168	StMYB017	StMYB168
StMYB182	StMYB020	StMYB182
	StMYB024	
	StMYB028	
	StMYB029	
	StMYB030	
	StMYB032	
	StMYB033	
	StMYB034	
	StMYB035	
	StMYB036	
	StMYB041	
	StMYB047	
	StMYB052	
	StMYB053	
	StMYB055	
	StMYB056	
	StMYB057	
	StMYB058	
	StMYB059	
	StMYB060	
	StMYB064	
	StMYB065	
	StMYB074	
	StMYB076	
	StMYB077	
	StMYB080	
	StMYB081	
	StMYB082	

StMYB083
StMYB094
StMYB099
StMYB108
StMYB109
StMYB110
StMYB112
StMYB117
StMYB118
StMYB123
StMYB124
StMYB128
StMYB129
StMYB130
StMYB132
StMYB137
StMYB145
StMYB147
StMYB151
StMYB154
StMYB156
StMYB159
StMYB160
StMYB161
StMYB162
StMYB168
StMYB175
StMYB180
StMYB181
StMYB182
StMYB187
StMYB193
StMYB194
StMYB197
StMYB205
StMYB209
StMYB212
StMYB213
StMYB214
StMYB221
StMYB226
StMYB230

Table S4. The detail information of segmental and tandem duplication gene pairs.

Duplicated pairs	Ka	Ks	Ka/Ks	Purifying selection	Duplicate type
StMYB001-StMYB127	0.231657866	0.533981551	0.433831218	Yes	Segmental
StMYB005-StMYB108	0.320887594	0.987543965	0.324934996	Yes	Segmental
StMYB015-StMYB079	0.436948939	1.431285292	0.305284308	Yes	Segmental
StMYB016-StMYB080	0.362370396	0.915456049	0.39583593	Yes	Segmental
StMYB017-StMYB180	0.149211058	0.684645148	0.217939262	Yes	Segmental
StMYB021-StMYB034	0.216430557	0.941455574	0.229889294	Yes	Segmental
StMYB021-StMYB123	0.427343742	1.570669003	0.272077529	Yes	Segmental
StMYB022-StMYB038	0.334963535	0.955290081	0.35064065	Yes	Segmental
StMYB024-StMYB144	0.338592841	2.556084146	0.132465452	Yes	Segmental
StMYB028-StMYB080	0.257435289	1.494765232	0.172224563	Yes	Segmental
StMYB029-StMYB076	0.49739006	1.808415932	0.275041848	Yes	Segmental
StMYB030-StMYB077	0.317739206	2.648134274	0.119986063	Yes	Segmental
StMYB033-StMYB087	0.413991779	2.102078373	0.196944027	Yes	Segmental
StMYB036-StMYB095	0.330767471	3.179463382	0.104032483	Yes	Segmental
StMYB037-StMYB118	0.24358666	0.667620486	0.364857977	Yes	Segmental
StMYB037-StMYB168	0.272243901	0.743699125	0.366067261	Yes	Segmental
StMYB050-StMYB126	0.200303211	0.512278387	0.39100461	Yes	Segmental
StMYB053-StMYB124	0.225116134	0.819136049	0.274821422	Yes	Segmental
StMYB055-StMYB123	0.201867967	0.952397188	0.211957752	Yes	Segmental
StMYB057-StMYB083	0.39592873	2.022416866	0.195770089	Yes	Segmental
StMYB109-StMYB128	0.231116446	1.005434381	0.229867259	Yes	Segmental
StMYB119-StMYB154	0.175167534	0.596289028	0.293762799	Yes	Segmental
StMYB175-StMYB145	0.263602546	0.938642525	0.280833799	Yes	Segmental
StMYB181-StMYB167	0.272800827	0.41578841	0.573302426	Yes	Segmental
StMYB182-StMYB129	0.309088601	0.41578841	0.743379549	Yes	Segmental
StMYB191-StMYB194	0.11029832	0.780224855	0.14136735	Yes	Segmental
StMYB197-StMYB106	0.128809831	1.122166423	0.114786745	Yes	Segmental
StMYB217-StMYB135	0.253608708	0.468470426	0.541354788	Yes	Segmental
StMYB218-StMYB137	0.300748484	0.751649735	0.400117861	Yes	Segmental
StMYB224-StMYB080	0.210957138	0.773501597	0.272730061	Yes	Segmental
StMYB230-StMYB103	0.30884068	0.693422348	0.445386108	Yes	Segmental
StMYB042-StMYB043	0.035558334	0.06385254	0.556882059	Yes	Tandem
StMYB087-StMYB088	0.045291753	0.229445291	0.197396742	Yes	Tandem
StMYB091-StMYB092	0.077931752	0.157172244	0.495836607	Yes	Tandem
StMYB110-StMYB111	0.104317981	0.389781518	0.267631933	Yes	Tandem
StMYB113-StMYB114	0.062492341	0.255720333	0.244377678	Yes	Tandem
StMYB114-StMYB115	0.046446093	0.134468056	0.345406145	Yes	Tandem
StMYB138-StMYB139	0.063018473	0.198023522	0.318237311	Yes	Tandem
StMYB139-StMYB140	0.093379154	0.235059319	0.397257823	Yes	Tandem
StMYB163-StMYB164	0.044841745	0.079216184	0.566067974	Yes	Tandem
StMYB227-StMYB228	0.025984659	0.164937952	0.157542025	Yes	Tandem

Table S5. The detail information of the induced *StMYB* gene under various stress treatments.

Stress treatment(s)	Induced gene(s)											
BABA	*StMYB155*	*StMYB128*										
BABA/Heat	*StMYB135*											
BABA/Heat/Mannitol/Salt	*StMYB118*											
BABA/Mannitol	*StMYB003*											
BABA/P. infestans	*StMYB022*											
BTH	*StMYB124*	*StMYB077*	*StMYB122*	*StMYB210*								
BTH/Heat/Mannitol/Salt	*StMYB017*	*StMYB024*										
BTH/Heat/P. infestans	*StMYB198*											
BTH/Heat/Salt/Wounding	*StMYB170*											
BTH/Mannitol	*StMYB101*											
BTH/Mannitol/Salt	*StMYB102*											
BTH/Salt	*StMYB005*	*StMYB096*										
Heat	*StMYB100*	*StMYB094*	*StMYB132*	*StMYB008*	*StMYB176*	*StMYB076*	*StMYB084*	*StMYB183*	*StMYB181*	*StMYB169*	*StMYB107*	*StMYB105*
Heat/Mannitol	*StMYB018*											
Heat/Mannitol/Salt	*StMYB037*	*StMYB144*	*StMYB168*	*StMYB166*	*StMYB066*	*StMYB063*	*StMYB212*					
Heat/Mannitol/Salt/Wounding	*StMYB112*	*StMYB110*	*StMYB080*									
Heat/Mannitol/Wounding	*StMYB048*											
Heat/Salt	*StMYB190*	*StMYB172*	*StMYB229*	*StMYB180*	*StMYB193*	*StMYB167*						
Heat/Salt/P. infestans	*StMYB162*											
Heat/Salt/Wounding	*StMYB111*											
Heat/Wounding	*StMYB171*	*StMYB145*	*StMYB186*	*StMYB175*	*StMYB069*							
Mannitol	*StMYB197*	*StMYB221*	*StMYB012*	*StMYB014*	*StMYB082*	*StMYB185*	*StMYB086*	*StMYB106*				
Mannitol/Salt	*StMYB133*	*StMYB007*	*StMYB150*	*StMYB055*	*StMYB030*	*StMYB129*	*StMYB010*	*StMYB081*	*StMYB147*	*StMYB120*	*StMYB021*	*StMYB060*
Mannitol/Salt/Wounding	*StMYB151*	*StMYB116*										
Mannitol/Wounding	*StMYB016*											
P. infestans	*StMYB225*											
Salt	*StMYB178*	*StMYB013*	*StMYB050*	*StMYB208*	*StMYB085*	*StMYB189*						
Wounding	*StMYB123*	*StMYB058*	*StMYB231*									

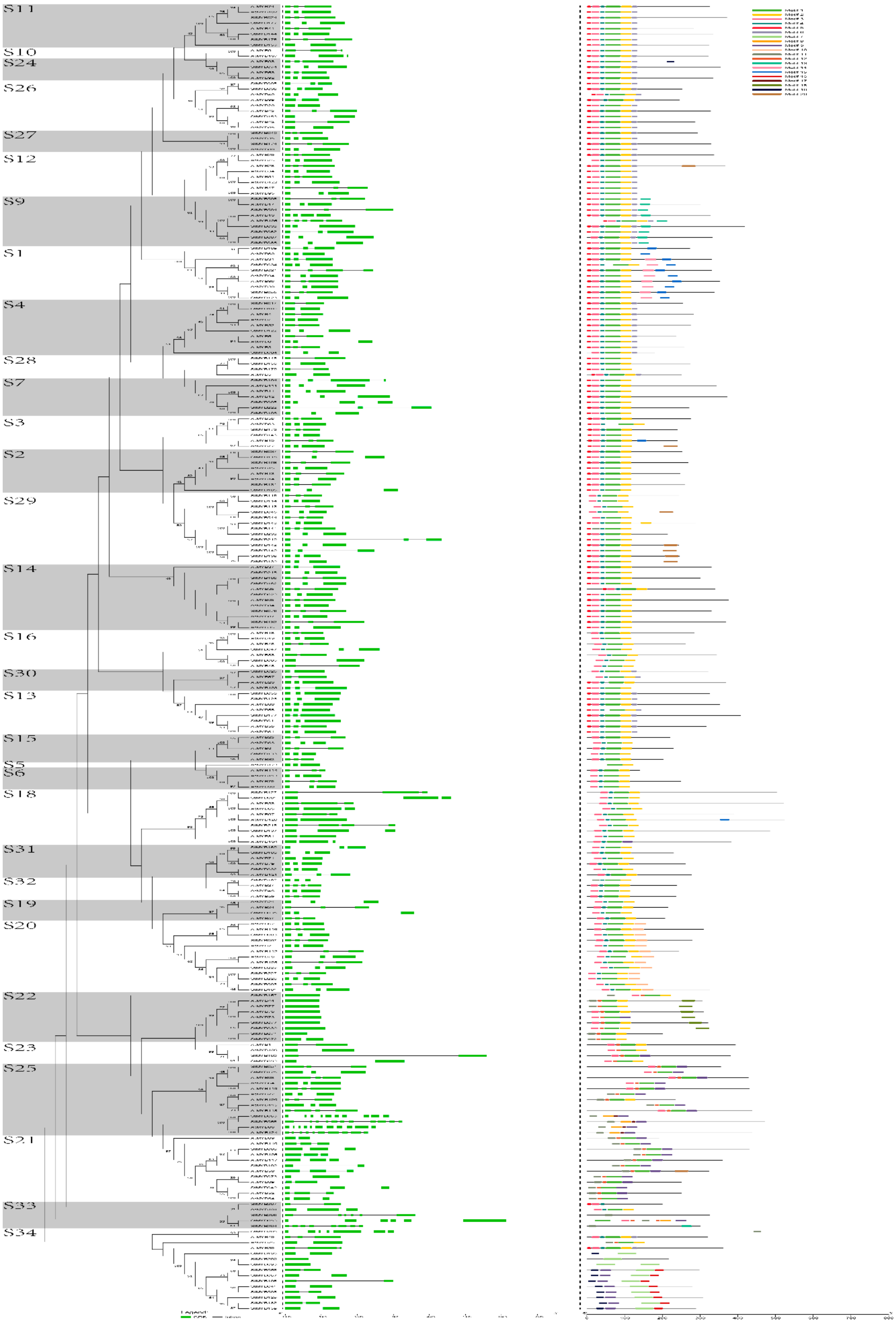

Figure S1**.** The motif and gene structure organizations of StMYB members.

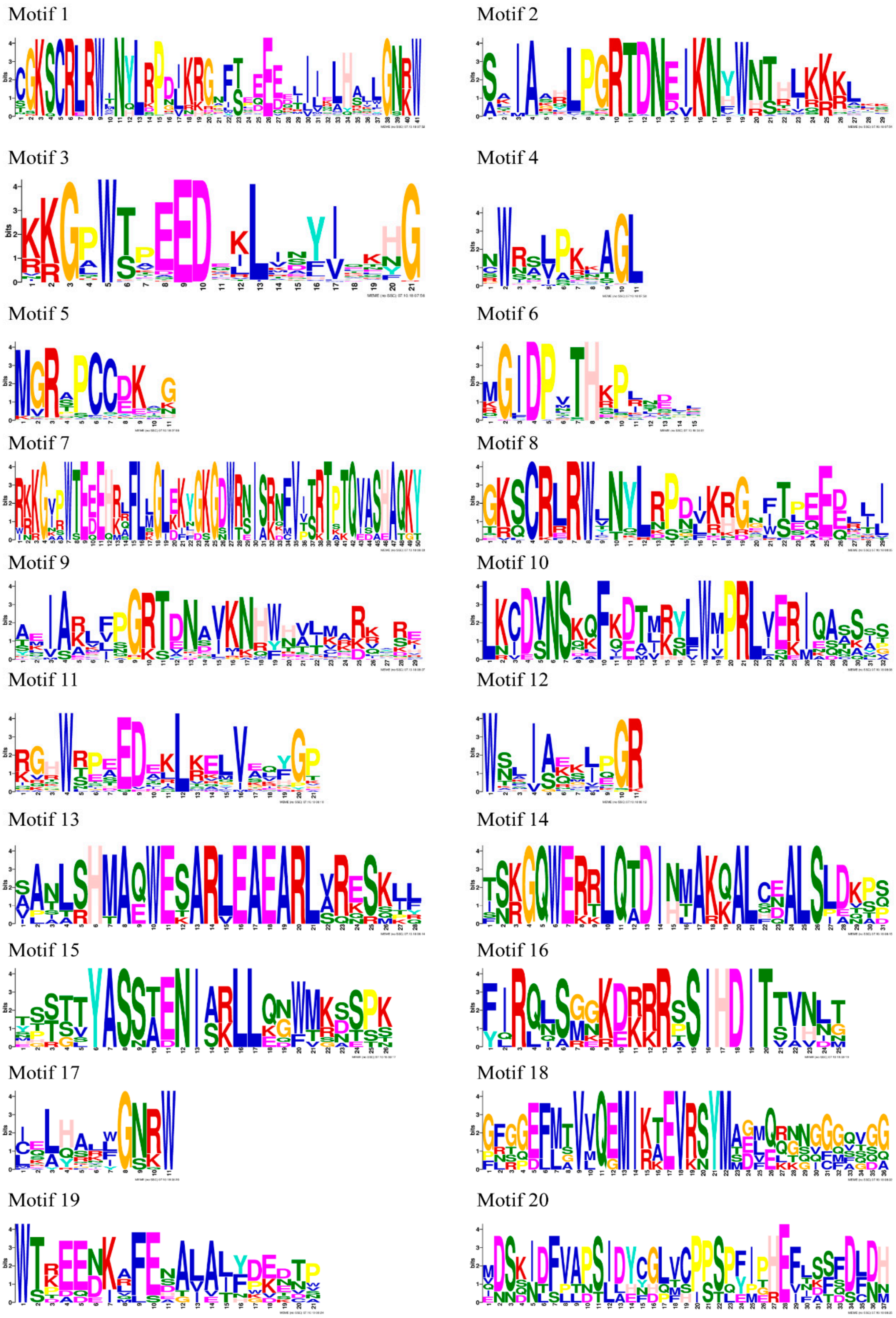

Figure S2. The putative conserved motifs in StMYB proteins.

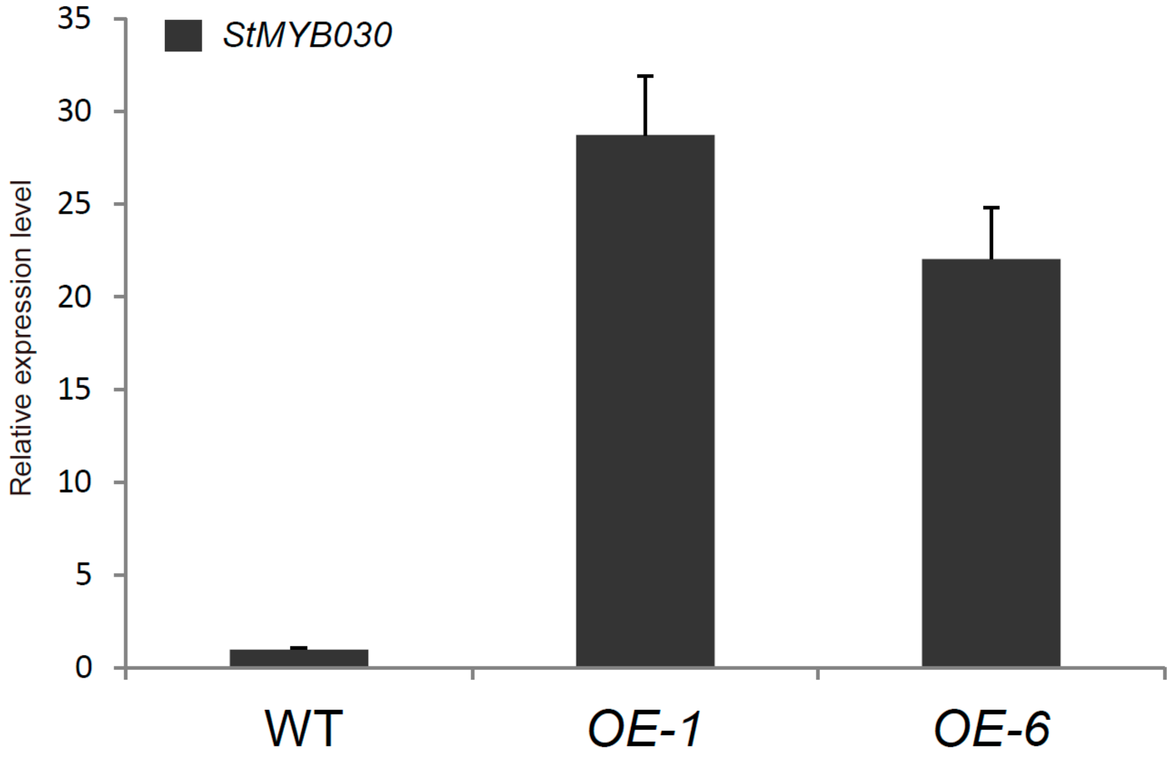

Figure S3. The expression level of *StMYB030* gene in wildtype and two overexpression lines, the ratios of gene expression level were calculated relative to the wildtype.
